# Understanding the determinants of COVID-19 vaccination intention and willingness to pay: findings from a population-based survey in Bangladesh

**DOI:** 10.1186/s12879-021-06406-y

**Published:** 2021-08-31

**Authors:** Rajon Banik, Md. Saiful Islam, Mamun Ur Rashid Pranta, Quazi Maksudur Rahman, Mahmudur Rahman, Shahina Pardhan, Robin Driscoll, Sahadat Hossain, Md. Tajuddin Sikder

**Affiliations:** 1 Department of Public Health and Informatics, Jahangirnagar University, Savar, Dhaka, 1342 Bangladesh; 2 Centre for Advanced Research Excellence in Public Health, Savar, Dhaka, 1342 Bangladesh; 3 Vision and Eye Research Institute, School of Medicine, Anglia Ruskin University, Young Street, Cambridge, UK

**Keywords:** COVID-19 vaccine, Health belief model, Intention, Willingness to pay, Bangladesh

## Abstract

**Background:**

Several coronavirus disease (COVID-19) vaccines have already been authorized and distributed in different countries all over the world, including Bangladesh. Understanding public acceptance of such a novel vaccine is vital, but little is known about the topic.

**Objectives:**

This study aimed to investigate the determinants of intention to receive a COVID-19 vaccine and willingness to pay (WTP) among people in Bangladesh.

**Methods:**

An anonymous and online-based survey of Bangladeshi people (mean age = 29.96 ± 9.15 years; age range = 18–60 years) was conducted using a self-reported questionnaire consisting of socio-demographics, COVID-19 experience, and vaccination-related information as well as the health belief model (HBM). Multivariable logistic regression was performed to determine the factors influencing COVID-19 vaccination intent and WTP.

**Results:**

Of the 894 participants, 38.5% reported a definite intention to receive a COVID-19 vaccine, whereas 27% had a probable intention, and among this intent group, 42.8% wanted to get vaccinated as soon as possible. Older age, feeling optimistic about the effectiveness of COVID-19 vaccination, believing that vaccination decreases worries and risk of COVID-19 infection, and being less concerned about side effects and safety of COVID-19 vaccination under the HBM construct were found to be significant factors in COVID-19 vaccination intention. Most of the participants (72.9%) were willing to pay for a COVID-19 vaccine, with a median (interquartile range [IQR]) amount of BDT 400/US$ 4.72 (IQR; BDT 200–600/US$ 2.36–7.07) per dose. Factors associated with higher WTP were younger age, being male, having higher education, residing in an urban area, having good self-rated health status, positivity towards COVID-19 vaccination's effectiveness, and being worried about the likelihood of getting infected with COVID-19. Participants who were COVID-19 vaccination intent preferred an imported vaccine over a domestically-made vaccine (22.9% vs. 14.8%), while 28.2% preferred a routine immunization schedule.

**Conclusion:**

The findings indicate a considerable proportion of Bangladeshi people intended to get vaccinated and had WTP for the COVID-19 vaccine. However, urgent education and awareness programs are warranted to alleviate public skepticism regarding the COVID-19 vaccination.

**Supplementary Information:**

The online version contains supplementary material available at 10.1186/s12879-021-06406-y.

## Introduction

The coronavirus disease 2019 (COVID-19), which emerged in Wuhan, Hubei Province, China at the end of 2019, has caused a large global outbreak and has become a major public health crisis [1, 2]. COVID-19 is a highly transmittable viral infection caused by a novel strain of severe acute respiratory syndrome coronavirus 2 (SARS-CoV-2) [3]. On March 11, 2020, the World Health Organization (WHO) declared the emergence of COVID-19 as a pandemic [4] which has affected more than 172 million people worldwide [5]. In Bangladesh, approximately 802,305 confirmed cases of COVID-19 were reported as of June 1, 2021, with a death toll of 12,660 [6]. This pandemic has severely affected people’s physical and psychological well-being [7–11], health system [12, 13] and also caused a major global economic recession [14].

Vaccines are the most effective strategy to protect the population from the devastating outcomes of COVID-19 [15, 16]. More than 287 potential vaccines are being developed and over 102 clinical trials have recently been released [16, 17]. Some have shown positive results, leading to a number of countries approving specific vaccines for implementation in vaccination programs. Meanwhile, by June 1, 2021, over 1.9 billion doses of the COVID-19 vaccine had been administered in 231 locations [18]. Bangladesh began mass vaccination on February 8, 2021 [19]. Despite considerable progress towards the vaccination program, there is some hesitancy about the COVID-19 vaccine [20]. Understanding public perception is crucial in order to achieve high vaccination coverage, especially for newly emerging infectious diseases such as COVID-19 [21–23]. According to recent studies on public acceptance of COVID-19 vaccination, the intention to take the vaccine ranged from 67 to 91% across countries such as India, Saudi-Arabia, Canada, the United States, and China [24–29]. There are multiple factors that may influence people’s vaccination intentions. Several demographic factors and perception of the disease risk have been found to be significantly associated with COVID-19 vaccination intent [28–30]. The health belief model (HBM) is one of the most commonly used models to determine factors associated with vaccination intention [31, 32] and has been used in many previous studies [33–35]. The HBM comprises several main constructs: perceived susceptibility, severity, benefits, barriers, self-efficacy to engage in a behavior, and cues to action [31]. Perceived stigma is also used for identifying determinants of vaccination intent [25]. In terms of HBM, perceived benefits (i.e. decreasing the chance of infection and making people less worried about infection) and barriers (i.e., being concerned about their efficacy) to vaccination were found to be significant in affecting vaccination intention [35, 36]. In addition, attitudes and experience regarding vaccination history, and convenience have been shown to be the major predictors of vaccination intention [29, 30].

Willingness-to-pay (WTP) refers to the maximum amount, in monetary terms, that an individual would be willing to allocate to obtain the benefits of a program [37]. The decision to vaccinate depends on the WTP of an individual in order to obtain increased health benefits [38]. HBM constructs have been used to explain WTP for influenza vaccination [34, 39]. In a previous study, the WTP for COVID-19 vaccination was found to be influenced by a variety of socioeconomic factors [36]. In addition, no-affordability barriers [35], as well as being aware of the perceived risks associated with higher WTP [38]. More evidence around public acceptance and WTP for the COVID-19 vaccine is essential to evaluate the success of vaccination programs, and to provide insights into future pricing considerations and demand forecasts.

To date, no research has been carried out in Bangladesh on people’s acceptance of the COVID-19 vaccine, the WTP, and the influencing factors and obstacles to vaccination coverage. The current study is aimed at determining the intention and WTP for a COVID-19 vaccine and other associated factors among people in Bangladesh.

## Materials and methods

### Study design, participants, and sampling

A cross-sectional online-based survey was carried out between 10 December 2020 and 10 January 2021. The inclusion criteria for participating were age ≥ 18 years, social media users (Facebook, WhatsApp, etc.), and currently living in Bangladesh. Incomplete surveys, individuals below 18 years old, and those who did not consent to the survey were excluded. Participants were not awarded any incentives or remuneration for taking part, and all responses were anonymous.

### Study procedure

The study used an online survey tool (Google Forms) to collect data, which was advertised and disseminated across different social media platforms (Facebook, WhatsApp, etc.). Participants were asked, *“Are you willing to participate in this study voluntarily?”* with “yes/no” responses. If the response was positive, they were given access to the full questionnaire. Otherwise, a blank survey form was submitted automatically. The questionnaire was translated into Bangla (the native language of participants) and then translated back to English and pre-tested with 40 individuals before starting the final data collection for acceptability and clarity. A total of 1032 participants completed the online survey form where 894 participants were included in the final analysis, following quality control and manual check procedures to exclude incomplete and invalid surveys.

### Sampling method

The sample size was calculated using the following equation:

$$ n=\frac{z^2 pq}{d^2};n=\frac{1.96^2\times 0.5\times \left(1-0.5\right)}{0.05^2}=384.16\approx 384 $$

Here,

*n* = number of samples

*z* = 1.96 (95% confidence level)

*p* = prevalence estimate (0.5)

*q* = (1-*p*)

*d* = precision limit or proportion of sampling error (0.05)

Assuming a 10% non-response rate, a total of 423.5 ≈ 424 sample size was estimated. However, the final sample exceeded this estimate.

### Survey instruments

A self-reported semi-structured questionnaire was developed after reviewing previous studies on COVID-19 vaccine uptake [25, 29, 36]. The survey consisted of questions about (1) socio-demographic information, health status, COVID-19 experience, and vaccination-related information; (2) beliefs about COVID-19 infection and COVID-19 vaccination; (3) intention to receive the COVID-19 vaccine; (4) WTP for the COVID-19 vaccine; and (5) participant’s vaccine preference.

### Socio-demographic, health status, COVID-19 experience, and vaccination-related information

Participants’ details, including age, sex, marital status, education level, monthly family income, number of children in the family, and area of residence were recorded. Participants were also asked to rate their overall health status, and whether or not they had any existing chronic diseases. Participants responded to their experience regarding COVID-19, whether or not they perceived COVID-19 vaccination as an effective way to prevent and control COVID-19 and whether or not they perceived a doctor’s recommendation as an important factor for COVID-19 vaccination decision. Information about the history of any vaccine hesitancy was also obtained.

### Beliefs about COVID-19 infection and COVID-19 vaccination

Participants’ beliefs about COVID-19 infection and COVID-19 vaccination were measured using HBM [40]. The questions probed perceived stigma of COVID-19 (four items), perceived susceptibility to COVID-19 (three items), perceived severity of COVID-19 (three items), perceived benefits of COVID-19 vaccination (two items), perceived barriers to getting a vaccination against COVID-19 (five items), and cues to action (two items). All construct questions of the health belief model were measured on a 5-point Likert scale ranging from 1 (*strongly disagree*) to 5 (*strongly agree*) [35, 41]. For simplification, the responses were recoded as “agree” (strongly agree/agree) and “disagree” (strongly disagree/disagree/not sure) during the final analysis.

### Intention to receive a COVID-19 vaccine and willingness to pay

Participant’s intention to receive a COVID-19 vaccine was measured by asking *“If a vaccine against COVID-19 infection was available, would you be willing to take it*?” Response options included “definitely not,” “probably not,” “not sure,” “probably yes,” and “definitely yes.” For our primary outcome, we dichotomized these responses into “yes” (definitely/probably yes) or “no” (all other responses). To assess the WTP for a COVID-19 vaccine, the question was *“Would you be willing to pay out-of-pocket for a COVID-19 vaccine?”* with “yes/no” responses*.* Participants who responded positively (yes) were asked *“What is the maximum amount you are willing to pay for a dose of the COVID-19 vaccine?”* The response options for price per dose were based on a 10-point scale and ranged from BDT 100 (≈ US$ 1.18) to BDT 1000 (≈ US$ 11.79). One United States Dollar (US$) is equivalent to 84.81 Bangladeshi Taka (BDT).

### Participant’s vaccine preference

Participants were asked *“How soon would you like to receive a COVID-19 vaccine when it becomes available?”* with two response options: *“*I will receive the vaccine as soon as possible” or “I will delay”. This was then followed by a question, *“Which type of COVID-19 vaccine would you prefer?”* with response options: “domestically-made vaccine”, “imported vaccine” or “both are acceptable”. Lastly, participants were asked *"What kind of immunization schedule do you prefer for the COVID-19 vaccination?”* with response options: “routine immunization”, “emergency vaccination” or “both are acceptable".

### Statistical analysis

All statistical analyses were performed using IBM Statistical Package for the Social Sciences software (SPSS; version 25.0). Descriptive analyses, including frequencies, percentages, means, standard deviations, etc. were computed. Bivariate logistic regression analysis was performed on the unadjusted estimates. Variables that were significant (*p* < 0.05) in the bivariate logistic regression analysis were included in the adjusted multivariable logistic regression model. A *p*-value less than 0.05 was considered statistically significant.

## Results

### Socio-demographics

The sample comprised 894 survey responses. The participants’ age ranged from 18 to 60 years with a mean age of 29.96 (SD 9.15) years and approximately half of the participants were female (50.3%). About 57.2% of the participants were unmarried and 55.9% had a bachelor’s degree, 30.4% reported having a monthly family income of > 40,000 BDT and 78.3% resided in urban areas (Table 1).

**Table 1:** Distribution of all variables and their associations with the intention to receive a COVID-19 vaccine

Variables	Overall *N*=894	Intention to receive a COVID-19 vaccine					
		*No*^b^	*Yes*^a^	OR (95% CI)	*p*-value	aOR (95% CI)	*p*-value
	n (%)	n (%)	n (%)				
***Socio-demographics***							
**Age**							
18-25 years	328 (36.7)	107 (34.7)	221 (37.7)	Reference		Reference	
26-35 years	339 (37.9)	151 (49)	188 (32.1)	0.603 (0.44-0.826)	**0.002**	0.735 (0.502-1.075)	0.112
36-45 years	171 (19.1)	38 (12.3)	133 (22.7)	1.695 (1.104-2.6)	**0.016**	1.682 (1.032-2.742)	**0.037**
> 45 years	56 (6.3)	12 (3.9)	44 (7.5)	1.775 (0.9-3.5)	0.097	2.123 (0.936-4.815)	0.072
**Sex**							
Male	444 (49.7)	151 (49)	293 (50)	1.04 (0.789-1.37)	0.782	–	–
Female	450 (50.3)	157 (51)	293 (50)	Reference			
**Marital status**							
Unmarried	511 (57.2)	175 (56.8)	336 (57.3)	1.016 (0.764-1.351)	0.914	–	–
Married	357 (39.9)	121 (39.3)	236 (40.3)	0.608 (0.275-1.342)	0.218	–	–
Divorced	26 (2.9)	12 (3.9)	14 (2.4)	Reference			
**Education level**							
Bachelor	500 (55.9)	170 (55.2)	330 (56.3)	1.255 (0.913-1.723)	0.162	1.165 (0.787-1.726)	0.445
Master's and above	152 (17)	43 (14)	109 (18.6)	1.638 (1.058-2.537)	**0.027**	1.539 (0.908-2.606)	0.109
Intermediate or below	242 (27.1)	95 (30.8)	147 (25.1)	Reference		Reference	
**Monthly family income**							
< 20000 BDT	191 (21.4)	60 (19.5)	131 (22.4)	1.269 (0.857-1.88)	0.234	–	–
20000-30000 BDT	193 (21.6)	58 (18.8)	135 (23)	1.353 (0.912-2.007)	0.133	–	–
30000-40000 BDT	238 (26.6)	90 (29.2)	148 (25.3)	0.956 (0.667-1.37)	0.807	–	–
> 40000 BDT	272 (30.4)	100 (32.5)	172 (29.4)	Reference			
**Number of children in the family**							
0	443 (49.6)	152 (49.4)	291 (49.7)	1.436 (1.033-1.996)	**0.031**	1.486 (0.989-2.234)	0.057
1	227 (25.4)	60 (19.5)	167 (28.5)	2.087 (1.404-3.103)	**<0.001**	1.658 (1.046-2.627)	0.081
≥2	224 (25.1)	96 (31.2)	128 (21.8)	Reference		Reference	
**Place of residence**							
Urban	700 (78.3)	238 (77.3)	462 (78.8)	1.096 (0.786-1.528)	0.589	–	–
Rural	194 (21.7)	70 (22.7)	124 (21.2)	Reference			
***Health status, COVID-19 experience, and vaccination-related information***							
**Self-rated health status**							
Good	633 (70.8)	219 (71.1)	414 (70.6)	0.978 (0.722-1.325)	0.887	–	–
Poor	261 (29.2)	89 (28.9)	172 (29.4)	Reference			
**History of chronic disease**							
Yes	248 (27.7)	92 (29.9)	156 (26.6)	0.852 (0.628-1.156)	0.303	–	–
No	646 (72.3)	216 (70.1)	430 (73.4)	Reference			
**Ever tested for COVID-19**							
Yes	219 (24.5)	66 (21.4)	153 (26.1)	1.296 (0.933-1.8)	0.123	–	–
No	675 (75.5)	242 (78.6)	433 (73.9)	Reference			
**Ever diagnosed with COVID-19**							
Yes	167 (18.7)	56 (18.2)	111 (18.9)	1.052 (0.737-1.501)	0.782	–	–
No	727 (81.3)	252 (81.8)	475 (81.1)	Reference			
**Family member/friend ever infected by COVID-19**							
Yes	235 (26.3)	94 (30.5)	141 (24.1)	Reference		Reference	
No	659 (73.7)	214 (69.5)	445 (75.9)	1.386 (1.019-1.886)	**0.037**	1.21 (0.838-1.747)	0.309
**Impact of COVID-19 on daily life**							
Severe^c^	353 (39.5)	102 (33.1)	251 (42.8)	1.513 (1.134-2.019)	**0.005**	0.914 (0.593-1.408)	0.684
Little^d^	541 (60.5)	206 (66.9)	335 (57.2)	Reference		Reference	
**Impact of COVID-19 on studies/work**							
Severe^c^	470 (52.6)	136 (44.2)	334 (57)	1.676 (1.269-2.214)	**<0.001**	1.167 (0.762-1.787)	0.479
Little^d^	424 (47.4)	172 (55.8)	252 (43)	Reference		Reference	
**Impact of COVID-19 on physical/mental health**							
Severe^c^	398 (44.5)	110 (35.7)	288 (49.1)	1.74 (1.31-2.311)	**<0.001**	0.968 (0.639-1.466)	0.879
Little^d^	496 (55.5)	198 (64.3)	298 (50.9)	Reference		Reference	
**COVID-19 vaccination is an effective way to prevent and control COVID-19**							
Yes	704 (78.7)	188 (61)	516 (88.1)	4.705 (3.353-6.602)	**<0.001**	2.709 (1.827-4.015)	**<0.001**
No	190 (21.3)	120 (39)	70 (11.9)	Reference		Reference	
**Doctor’s recommendation is an important factor in vaccination decision-making**							
Yes	797 (89.1)	253 (82.1)	544 (92.8)	2.816 (1.835-4.322)	**<0.001**	1.579 (0.935-2.664)	0.087
No	97 (10.9)	55 (17.9)	42 (7.2)	Reference		Reference	
**Previous refusals to get any type of vaccination**							
No	728 (81.4)	241 (78.2)	487 (83.1)	1.368 (0.967-1.934)	0.076	–	–
Yes	166 (18.6)	67 (21.8)	99 (16.9)	Reference			
***Perceived stigma of COVID-19***							
**If I had COVID-19, I would be embarrassed**							
Agree^e^	232 (26)	63 (20.5)	169 (28.8)	1.576 (1.134-2.191)	**0.007**	1.117 (0.688-1.815)	0.654
Disagree^f^	662 (74)	245 (79.5)	417 (71.2)	Reference		Reference	
**If I had COVID-19, people would think badly of me**							
Agree^e^	230 (25.7)	63 (20.5)	167 (28.5)	1.55 (1.114-2.156)	**0.009**	0.695 (0.389-1.243)	0.220
Disagree^f^	664 (74.3)	245 (79.5)	419 (71.5)	Reference		Reference	
**If I had COVID-19, people would treat me differently.**							
Agree^e^	276 (30.9)	72 (23.4)	204 (34.8)	1.75 (1.279-2.396)	**<0.001**	1.561 (0.917-2.657)	0.101
Disagree^f^	618 (69.1)	236 (76.6)	382 (65.2)	Reference		Reference	
**If I had COVID-19, I would not tell anyone**							
Agree^e^	142 (15.9)	49 (15.9)	93 (15.9)	0.997 (0.684-1.454)	0.988	–	–
Disagree^f^	752 (84.1)	259 (84.1)	493 (84.1)	Reference			
***Perceived susceptibility of contracting COVID-19***							
**My chance of getting COVID-19 in the next few months is high**							
Agree^e^	226 (25.3)	71 (23.1)	155 (26.5)	1.2 (0.87-1.657)	0.267	–	–
Disagree^f^	668 (74.7)	237 (76.9)	431 (73.5)	Reference			
**I am worried about the likelihood of getting COVID 19**							
Agree^e^	406 (45.4)	112 (36.4)	294 (50.2)	1.762 (1.328-2.338)	**<0.001**	1.643 (1.065-2.537)	**0.025**
Disagree^f^	488 (54.6)	196 (63.6)	292 (49.8)	Reference		Reference	
**Getting COVID-19 is currently a possibility for me**							
Agree^e^	297 (33.2)	93 (30.2)	204 (34.8)	1.235 (0.918-1.661)	0.164	–	–
Disagree^f^	597 (66.8)	215 (69.8)	382 (65.2)	Reference			
***Perceived severity of COVID-19***							
**Complications from COVID-19 are serious**							
Agree^e^	412 (46.1)	120 (39)	292 (49.8)	1.556 (1.175-2.06)	**0.002**	0.997 (0.649-1.531)	0.987
Disagree^f^	482 (53.9)	188 (61)	294 (50.2)	Reference		Reference	
**I will be very sick if I get infected with COVID-19**							
Agree^e^	365 (40.8)	104 (33.8)	261 (44.5)	1.575 (1.182-2.099)	**0.002**	0.946 (0.606-1.478)	0.808
Disagree^f^	529 (59.2)	204 (66.2)	325 (55.5)	Reference		Reference	
**I will be very afraid if I become infected with COVID-19**							
Agree^e^	401 (44.9)	122 (39.6)	279 (47.6)	1.386 (1.047-1.833)	**0.022**	0.871 (0.555-1.366)	0.547
Disagree^f^	493 (55.1)	186 (60.4)	307 (52.4)	Reference		Reference	
***Perceived benefits of COVID-19 vaccination***							
**Vaccination is a good idea because I feel less worried about catching COVID-19**							
Agree^e^	329 (36.8)	46 (14.9)	283 (48.3)	5.32 (3.738-7.57)	**<0.001**	2.351 (1.385-3.988)	**0.002**
Disagree^f^	565 (63.2)	262 (85.1)	303 (51.7)	Reference		Reference	
**Vaccination decreases my chance of getting COVID-19 or its complications**							
Agree^e^	378 (42.3)	59 (19.2)	319 (54.4)	5.042 (3.636-6.993)	**<0.001**	3.083 (1.829-5.198)	**<0.001**
Disagree^f^	516 (57.7)	249 (80.8)	267 (45.6)	Reference		Reference	
***Perceived barriers of COVID-19 vaccination***							
**I am worried about the possible side effects of COVID-19 vaccination would interfere with my usual activities**							
Agree^e^	479 (53.6)	167 (54.2)	312 (53.2)	0.842 (0.689-1.072)	**0.060**	0.284 (0.216-0.561)	**0.002**
Disagree^f^	415 (46.4)	141 (45.8)	274 (46.8)	Reference		Reference	
**I am concerned about the efficacy of the COVID-19 vaccination**							
Agree^e^	470 (52.6)	164 (53.2)	306 (52.2)	0.96 (0.728-1.265)	0.770	–	–
Disagree^f^	424 (47.4)	144 (46.8)	280 (47.8)	Reference			
**I am concerned about the safety of the COVID-19 vaccination**							
Agree^e^	483 (54)	183 (59.4)	300 (51.2)	0.716 (0.542-0.947)	**0.019**	0.284 (0.187-0.429)	**<0.001**
Disagree^f^	411 (46)	125 (40.6)	286 (48.8)	Reference		Reference	
**I am concerned about the affordability (high cost of the vaccine) of getting the COVID-19 vaccination**							
Agree^e^	450 (50.3)	151 (49)	299 (51)	1.083 (0.822-1.427)	0.570	–	–
Disagree^f^	444 (49.7)	157 (51)	287 (49)	Reference			
**I am concerned about faulty/fake COVID-19 vaccines**							
Agree^e^	539 (60.3)	188 (61)	351 (59.9)	0.953 (0.719-1.264)	0.740	–	–
Disagree^f^	355 (39.7)	120 (39)	235 (40.1)	Reference			
***Cues to action***							
**I will only take the COVID-19 vaccine if I am given adequate information about it**							
Agree^e^	601 (67.2)	176 (57.1)	425 (72.5)	1.98 (1.482-2.645)	**<0.001**	1.273 (0.787-2.058)	0.325
Disagree^f^	293 (32.8)	132 (42.9)	161 (27.5)	Reference		Reference	
**I will only take the COVID-19 vaccine if the vaccine is taken by many in the public**							
Agree^e^	505 (56.5)	144 (46.8)	361 (61.6)	1.827 (1.382-2.415)	**<0.001**	0.878 (0.571-1.35)	0.553
Disagree^f^	389 (43.5)	164 (53.2)	225 (38.4)	Reference		Reference	

*OR* Odds Ratio, *CI* Confidence Interval, *aOR* Adjusted Odds Ratios, *BDT* Bangladeshi Taka

^a^Definitely yes/ probably yes

^b^Definitely no/ probably no/ not sure

^c^Very severe/ severe

^d^Very little/ little/ fair

^e^Strongly agree/ agree

^f^Strongly disagree/ disagree/ not sure

While the majority of participants reported good health status (70.8%), 27.7% reported having chronic underlying diseases. 18.7% of participants reported having already been diagnosed with COVID-19. More than a quarter of participants (26.3%) responded that their family members had been infected with COVID-19. The majority (89.1%) of participants perceived the doctor’s recommendation as an important factor in their decision to have the COVID-19 vaccine. While 18.6% reported previous vaccine hesitancy (Table 1).

### Health beliefs

The distribution of each item of the HBM is presented in Table 1. Approximately 15.9–30.9% agreed with regard to each construct-related stigma of COVID-19. With regards to the perceived susceptibility of contracting COVID-19, 74.7% of respondents disagreed that they had the possibility of contracting COVID-19 in the next few months; 45.4% were concerned about contracting COVID-19, and 33.2% thought that contracting COVID-19 was currently a possibility. Responses to questions about the perceived severity of COVID-19 demonstrate that less than half of respondents (46.1%) thought that complications of COVID-19 were serious and they would be very sick if they contracted COVID-19 (40.8%), or were afraid of contracting COVID-19 (44.9%). While the majority (78.7%) of participants perceived that vaccination was an effective way to prevent and control COVID-19, very few (36.8%) agreed that vaccination would make them feel less worried about contracting COVID-19, and vaccination would decrease their chance of contracting COVID-19 or its complications (42.3%). With regards to perceived barriers to COVID-19 vaccination, the majority of respondents (50.3–60.3%) had concerns about COVID-19 vaccination, including the impact of side-effects on usual activities (53.6%), efficacy (52.6%), safety (54%), affordability (50.3%), and validity (60.3%). In the cues to action section of the survey, over two-thirds of respondents confirmed that they would only take a vaccine if they were provided with adequate information (67.2%) and 43.5% disagreed with taking the COVID-19 vaccine if the vaccine was not taken by many in the public.

### COVID-19 vaccination intent

**Fig. 1:**
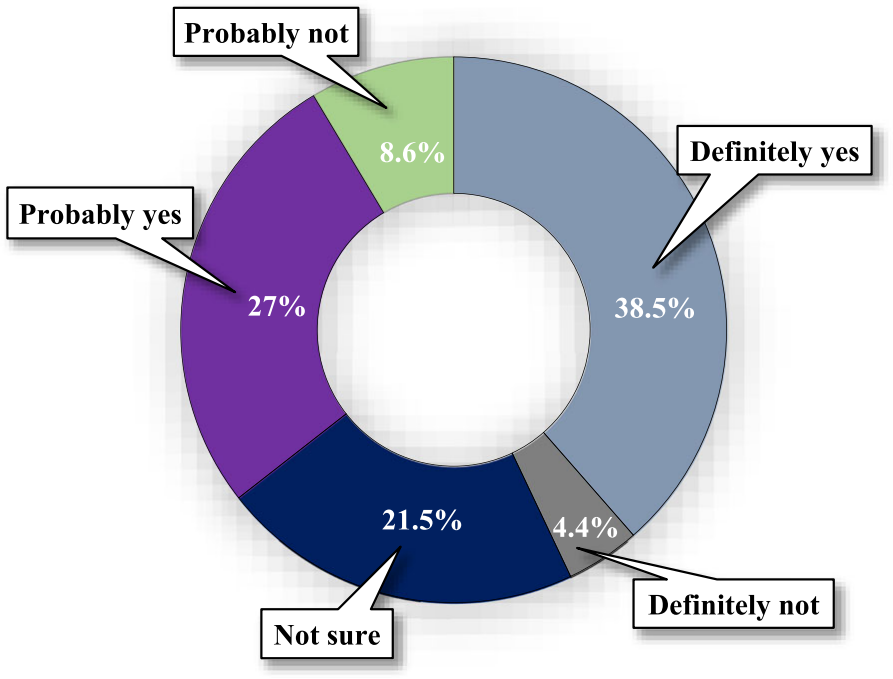
COVID-19 vaccination intent (*N* = 894)

Overall, 65.5% of participants reported a positive intention to receive a COVID-19 vaccine (38.5% definitely yes, and 27.0% probably yes); whilst 34.5% were unwilling or hesitant to be vaccinated against COVID-19 (21.5% not sure, 8.6% probably not, and 4.4% definitely not; Fig. 1). The results of bivariate and multivariable logistic regression of the intention to receive the vaccine are presented in Table 1. Bivariate analysis showed that the intention to receive the vaccine was significantly (*p* < 0.05) associated with being older, having higher education, having fewer children, having family members not infected with COVID-19, the severe impact of COVID-19 on participant's daily lives, studies/work and physical/mental health, positivity towards COVID-19 vaccination's effectiveness, and perceiving the doctor's recommendation as an important factor in vaccination decision making (Table 1). Multiple logistic regression, using only those variables that were significant in bivariate analysis, retained older age, positivity towards the effectiveness of COVID-19 vaccination, worries about the likelihood of being infected, believing that vaccination will safeguard against catching COVID-19 and decrease the risk of being infected with COVID-19 or its complications, and being less aware of the side-effects and safety of the COVID-19 vaccine (Table 1).

### Willingness to pay (WTP)

**Fig. 2:**
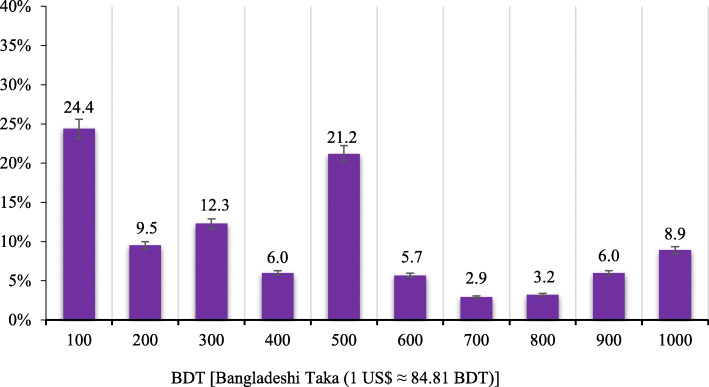
Willingness to pay for the COVID-19 vaccine (*N* = 652)

Almost three-quarters of participants (72.9%) were willing to pay for the COVID-19 vaccine. The median (interquartile range [IQR]) WTP of the willing group was BDT400/US$ 4.72 (IQR; BDT 200–600/US$ 2.35–7.07) per dose (Fig. 2). Bivariate analysis showed that WTP was significantly (*p* < 0.05) associated with being young, male, being single, having higher education, urban residency, having good self-rated health status, having no chronic underlying diseases, positivity towards the effectiveness of COVID-19 vaccination, perceiving the doctor's recommendation as an important factor in vaccination decision making, being worried about the likelihood of contracting COVID 19, believing that vaccination decreases the chance of contracting COVID-19 or if infected, its complications, and perception of being vaccinated if given enough information about the COVID-19 vaccine (Table 2). Figure 2 represents the amount of money participants WTP for the COVID-19 vaccine. Multiple logistic regression, using only those variables that were significant in bivariate analysis, retained younger age, male, higher education, urban resident, having good self-rated health status, positivity towards the effectiveness of COVID-19 vaccination, and being worried about the likelihood of contracting COVID-19 (Table 2).

**Table 2:** Distribution of all studied variables and their associations with the willingness to pay for a COVID-19 vaccine

Variables	Willingness to pay for a COVID-19 vaccine					
	*No* n (%)	*Yes* n (%)	OR (95% CI)	*p*-value	aOR (95% CI)	*p*-value
***Socio-demographics***						
**Age**						
18-25 years	111 (45.9)	217 (33.3)	1.173 (0.652-2.11)	0.594	1.148 (0.596-2.211)	0.680
26-35 years	73 (30.2)	266 (40.8)	2.186 (1.2-3.983)	**0.011**	2.068 (1.072-3.99)	**0.030**
36-45 years	37 (15.3)	134 (20.6)	2.173 (1.132-4.171)	**0.02**	1.715 (0.847-3.472)	0.134
> 45 years	21 (8.7)	35 (5.4)	Reference		Reference	
**Sex**						
Male	107 (44.2)	337 (51.7)	1.35 (1.003-1.816)	**0.047**	1.439 (1.044-1.985)	**0.026**
Female	135 (55.8)	315 (48.3)	Reference		Reference	
**Marital status**						
Unmarried	126 (52.1)	385 (59)	3.565 (1.607-7.909)	**0.002**	2.057 (0.847-4.993)	0.111
Married	102 (42.1)	255 (39.1)	2.917 (1.305-6.521)	0.159	2.299 (0.953-5.545)	0.064
Divorced	14 (5.8)	12 (1.8)	Reference		Reference	
**Education level**						
Bachelor	112 (46.3)	388 (59.5)	2.162 (1.549-3.018)	**<0.001**	1.701 (1.169-2.476)	**0.006**
Master's and above	37 (15.3)	115 (17.6)	1.94 (1.234-3.049)	**0.004**	1.414 (0.859-2.327)	0.173
Intermediate or below	93 (38.4)	149 (22.9)	Reference		Reference	
**Monthly family income**						
< 20000	58 (24)	133 (20.4)	0.779 (0.516-1.177)	0.236	–	–
20000-30000	53 (21.9)	140 (21.5)	0.898 (0.591-1.363)	0.613	–	–
30000-40000	62 (25.6)	176 (27)	0.965 (0.648-1.437)	0.86	–	–
> 40000	69 (28.5)	203 (31.1)	Reference			
**Number of children in the family**						
0	124 (51.2)	319 (48.9)	1.029 (0.72-1.47)	0.875	–	–
1	54 (22.3)	173 (26.5)	1.281 (0.841-1.953)	0.248	–	–
≥2	64 (26.4)	160 (24.5)	Reference			
**Place of residence**						
Urban	168 (69.4)	532 (81.6)	1.953 (1.393-2.737)	**<0.001**	1.687 (1.16-2.454)	**0.006**
Rural	74 (30.6)	120 (18.4)	Reference		Reference	
***Health status, COVID-19 experience, and vaccination-related information***						
**Self-rated health status**						
Good	146 (60.3)	487 (74.7)	1.941 (1.42-2.652)	**<0.001**	1.713 (1.215-2.417)	**0.002**
Poor	96 (39.7)	165 (25.3)	Reference		Reference	
**History of chronic disease**						
No	162 (66.9)	484 (74.2)	1.423 (1.033-1.96)	**0.031**	1.187 (0.817-1.724)	0.369
Yes	80 (33.1)	168 (25.8)	Reference		Reference	
**Ever tested for COVID-19**						
Yes	61 (25.2)	158 (24.2)	0.949 (0.675-1.335)	0.764	–	–
No	181 (74.8)	494 (75.8)	Reference			
**Ever diagnosed with COVID-19**						
Yes	46 (19)	121 (18.6)	0.971 (0.666-1.415)	0.878	–	–
No	196 (81)	531 (81.4)	Reference			
**Family member/friend ever infected by COVID-19**						
Yes	70 (28.9)	165 (25.3)	0.833 (0.599-1.157)	0.275	–	–
No	172 (71.1)	487 (74.7)	Reference			
**Impact of COVID-19 on daily life**						
Severe^a^	90 (37.2)	263 (40.3)	1.142 (0.842-1.548)	0.392	–	–
Little^b^	152 (62.8)	389 (59.7)	Reference			
**Impact of COVID-19 on studies/work**						
Severe^a^	122 (50.4)	348 (53.4)	1.126 (0.838-1.513)	0.431	–	–
Little^b^	120 (49.6)	304 (46.6)	Reference			
**Impact of COVID-19 on physical/mental health**						
Severe^a^	100 (41.3)	298 (45.7)	1.195 (0.887-1.611)	0.242	–	–
Little^b^	142 (58.7)	354 (54.3)	Reference			
**COVID-19 vaccination is an effective way to prevent and control COVID-19**						
Yes	161 (66.5)	543 (83.3)	2.506 (1.789-3.511)	**<0.001**	2.172 (1.486-3.176)	**<0.001**
No	81 (33.5)	109 (16.7)	Reference			
**Doctor’s recommendation is an important factor in vaccination decision-making**						
Yes	200 (82.6)	597 (91.6)	2.279 (1.479-3.512)	**<0.001**	1.549 (0.938-2.557)	0.087
No	42 (17.4)	55 (8.4)	Reference		Reference	
**Previous refusals to get any type of vaccination**						
No	192 (79.3)	536 (82.2)	1.203 (0.831-1.743)	0.327	–	–
Yes	50 (20.7)	116 (17.8)	Reference			
***Perceived stigma of COVID-19***						
**If I had COVID-19, I would be embarrassed**						
Agree^c^	64 (26.4)	168 (25.8)	0.965 (0.69-1.35)	0.837	–	–
Disagree^d^	178 (73.6)	484 (74.2)	Reference			
**If I had COVID-19, people would think badly of me**						
Agree^c^	66 (27.3)	164 (25.2)	0.896 (0.642-1.251)	0.520	–	–
Disagree^d^	176 (72.7)	488 (74.8)	Reference			
**If I had COVID-19, people would treat me differently.**						
Agree^c^	80 (33.1)	196 (30.1)	0.87 (0.635-1.194)	0.389	–	–
Disagree^d^	162 (66.9)	456 (69.9)	Reference			
**If I had COVID-19, I would not tell anyone**						
Agree^c^	46 (19)	96 (14.7)	0.736 (0.499-1.084)	0.120	–	–
Disagree^d^	196 (81)	556 (85.3)	Reference			
***Perceived susceptibility of contracting COVID-19***						
**My chance of getting COVID-19 in the next few months is high**						
Agree^c^	61 (25.2)	165 (25.3)	1.005 (0.716-1.412)	0.976	–	–
Disagree^d^	181 (74.8)	487 (74.7)	Reference			
**I am worried about the likelihood of getting COVID 19**						
Agree^c^	93 (38.4)	313 (48)	1.479 (1.095-1.999)	**0.011**	1.403 (1.001-1.967)	**0.049**
Disagree^d^	149 (61.6)	339 (52)	Reference		Reference	
**Getting COVID-19 is currently a possibility for me**						
Agree^c^	80 (33.1)	217 (33.3)	1.01 (0.738-1.382)	0.950	–	–
Disagree^d^	162 (66.9)	435 (66.7)	Reference			
***Perceived severity of COVID-19***						
**Complications from COVID-19 are serious**						
Agree^c^	123 (50.8)	289 (44.3)	0.77 (0.573-1.035)	0.084	–	–
Disagree^d^	119 (49.2)	363 (55.7)	Reference			
**I will be very sick if I get infected with COVID-19**						
Agree^c^	104 (43)	261 (40)	0.886 (0.657-1.194)	0.426	–	–
Disagree^d^	138 (57)	391 (60)	Reference			
**I will be very afraid if I become infected with COVID-19**						
Agree^c^	107 (44.2)	294 (45.1)	1.036 (0.77-1.394)	0.815	–	–
Disagree^d^	135 (55.8)	358 (54.9)	Reference			
***Perceived benefits of COVID-19 vaccination***						
**Vaccination is a good idea because I feel less worried about catching COVID-19**						
Agree^c^	78 (32.2)	251 (38.5)	1.316 (0.963-1.799)	0.085	–	–
Disagree^d^	164 (67.8)	401 (61.5)	Reference			
**Vaccination decreases my chance of getting COVID-19 or its complications**						
Agree^c^	85 (35.1)	293 (44.9)	1.507 (1.11-2.047)	**0.009**	1.15 (0.787-1.681)	0.47
Disagree^d^	157 (64.9)	359 (55.1)	Reference		Reference	
***Perceived barriers of COVID-19 vaccination***						
**I am worried the possible side-effects of COVID-19 vaccination would interfere with my usual activities**						
Agree^c^	112 (46.3)	303 (46.5)	1.008 (0.75-1.355)	0.959	–	–
Disagree^d^	130 (53.7)	349 (53.5)	Reference			
**I am concerned about the efficacy of the COVID-19 vaccination**						
Agree^c^	124 (51.2)	346 (53.1)	1.076 (0.801-1.446)	0.627	–	–
Disagree^d^	118 (48.8)	306 (46.9)	Reference			
**I am concerned about the safety of the COVID-19 vaccination**						
Agree^c^	127 (52.5)	356 (54.6)	1.089 (0.81-1.464)	0.572	–	–
Disagree^d^	115 (47.5)	296 (45.4)	Reference			
**I am concerned about the affordability (high cost of the vaccine) of getting the COVID-19 vaccination**						
Agree^c^	131 (54.1)	319 (48.9)	0.812 (0.604-1.091)	0.167	–	–
Disagree^d^	111 (45.9)	333 (51.1)	Reference			
**I am concerned about faulty/fake COVID-19 vaccines**						
Agree^c^	147 (60.7)	392 (60.1)	0.974 (0.72-1.318)	0.866	–	–
Disagree^d^	95 (39.3)	260 (39.9)	Reference			
***Cues to action***						
**I will only take the COVID-19 vaccine if I am given adequate information about it**						
Agree^c^	148 (61.2)	453 (69.5)	1.446 (1.063-1.966)	**0.019**	0.954 (0.643-1.415)	0.814
Disagree^d^	94 (38.8)	199 (30.5)	Reference		Reference	
**I will only take the COVID-19 vaccine if the vaccine is taken by many in the public**						
Agree^c^	125 (51.7)	380 (58.3)	1.308 (0.972-1.759)	0.076	–	–
Disagree^d^	117 (48.3)	272 (41.7)	Reference			

*OR* Odds Ratio, *CI* Confidence Interval, *aOR* Adjusted Odds Ratios, *BDT* Bangladeshi Taka

^a^Very severe/ severe

^b^Very little/ little/ fair

^c^Strongly agree/ agree

^d^Strongly disagree/ disagree/ not sure

### Vaccine preference

**Fig. 3:**
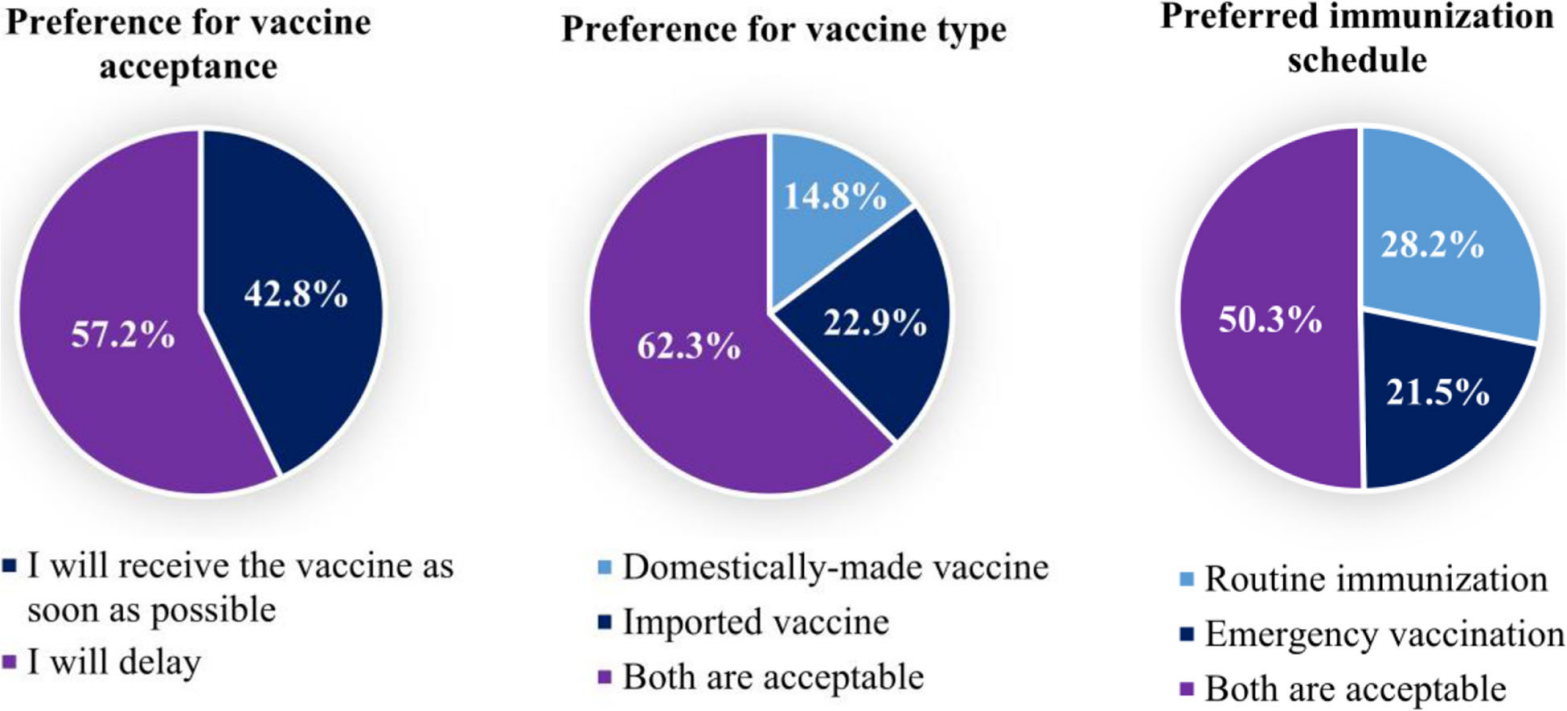
Vaccine preferences (*N* = 586)

Almost four in every ten participants who were COVID-19 vaccine intet reported that they would receive the vaccine as soon as possible (42.8%); whilst 57.2% reported that they would delay. 14.8% reported a domestically-made vaccine as their preference, 22.9% preferred an imported vaccine and 62.3% had no preference. In terms of immunization schedule, 28.2% preferred routine immunization, 21.5% an emergency vaccination schedule and 50.3% had no preference (Fig. 3).

## Discussion

Vaccines are a key solution to halting the escalation of pandemics such as COVID-19. The government of Bangladesh began the COVID-19 vaccination roll-out on February 8, 2021 [42]. As with any new vaccine, the COVID-19 vaccine raises concerns. The present study examined how likely people will be to take a COVID-19 vaccine and investigate whether people are willing to pay for it. Our finding represents one of the first estimates of the intention to receive the vaccine among Bangladeshi people and can be used to guide projections of future vaccine uptake and successful implementation of the COVID-19 vaccination program in Bangladesh.

In this study, the majority of participants (65.5%) reported a definite or probable intention to receive a COVID-19 vaccine, which is comparable with recent studies conducted in Saudi Arabia and the United States [25, 28]. A higher proportion of COVID-19 vaccine intention has been reported in similar studies conducted in China, India, Indonesia, and Malaysia, ranging from 83.5 to 94.3% [24, 27, 35, 36]. It may be possible that when the study was conducted, the outbreak of COVID-19 in Bangladesh was largely under control, and also there was a lack of adequate information about the vaccine. Participants in this study had a low level of perceived susceptibility to COVID-19, according to the HBM construct, which is consistent with previous studies [35, 36] and suggests that the Bangladeshi people were not aware of the possibility of the resurgence of COVID-19, making them feel less vulnerable. Our findings suggest that participants’ intention to receive a COVID-19 vaccine was dependent on various socio-demographic factors. In particular, older age was found to be a significant influential factor for the COVID-19 vaccine intention. This finding is justified by the fact that elderly people are at an increased risk of COVID-19 infection both in terms of its severity and also mortality [43]. Our findings highlight the need for education intervention focusing particularly on younger age groups. The participants’ education level was also found to be a significant factor in COVID-19 vaccine intention in the bivariate analysis, although it was not significant in the multivariate analysis. Similar results were shown in other earlier studies in Bangladesh, illustrating that individuals with a higher educational background had more knowledge and awareness regarding COVID-19 [44, 45].

The COVID-19 epidemic has had a significant impact on people all across the world, affecting work, income, and physical and mental health [46–48]. The present study found that having family members who had been infected and the perception of COVID-19’s impact on daily life, studies/work, and physical/mental health were significant factors in the bivariate analysis, agreeing with a recent study among Chinese citizens [29]. Majority of the study participants agreed that vaccination is an effective way to prevent and control COVID-19, and this was a significant factor for participant’s intention to receive a vaccine, agreeing with 89.5% of Chinese residents who thought that vaccination is an effective way to prevent and control COVID-19 [29]. This positive attitude towards COVID-19 vaccination and the significant impact that it would have on their life explains the intention to receive a vaccine among people in Bangladesh. Multivariable analysis found that vaccination intention was associated with participant’s beliefs [e.g., Health Belief Model (HBM)] towards COVID-19, consistent with previous studies [34, 36, 49]. In particular, our findings suggest that perceived susceptibility to being infected with COVID-19 and the perceived benefits of and barriers to COVID-19 vaccination are the most important HBM constructs influencing participants’ intention to receive a COVID-19 vaccine. Participants with high perceived susceptibility to being infected with COVID-19 expressed increased vaccination intention, consistent with previous studies [25, 35]. While less than half of the participants (45.4%) were worried about the likelihood of contracting COVID-19, relatively few (25.3%) perceived themselves as at high risk of becoming infected. This indicates the need to increase public education and awareness about risk, in order that preventive actions can be taken to improve COVID-19 pandemic control [50].

The findings of this study also suggest participants' lower perceived benefits of COVID-19 vaccination and relatively higher perceived barriers to getting COVID-19 vaccination. In contrast, a similar study conducted in China showed high perceived benefits and low perceived barriers towards COVID-19 vaccination among the participants [36]. This may be the reason why Bangladeshi people showed a lower intention to receive a COVID-19 vaccine compared to Malaysian and Chinese people [29, 35]. Public health intervention programs that focus on increasing awareness of the benefits of COVID-19 vaccination and reducing the identified barriers are therefore essential. The multivariate analysis found concern about the safety of the COVID-19 vaccination as a significant barrier to vaccination intention, with similar findings reported in other studies related to the new vaccine [51], suggesting that information regarding the safety and efficacy standards should be made available to the general public. Another significant barrier was the worry about possible side effects of the COVID-19 vaccine. Bangladesh has experienced various negative events associated with vaccine malpractices and scandals, which have resulted in the public losing confidence in the COVID-19 vaccines [52], which may be implied in this study, as a considerable proportion of reported concerns regarding the possibility of side-effects of COVID-19 vaccines.

This study revealed that the majority of participants (72.9%) were willing to pay for a COVID-19 vaccine. This finding is comparable with a recent study in Indonesia, which found 78.3% of participants had WTP for a COVID-19 vaccine [38]. Multivariate analysis found that WTP for a vaccine was significantly influenced by socio-demographic factors such as younger age, male sex, higher education level, and residing in an urban area. Younger people reported higher WTP for a COVID-19 vaccine, consistent with a recent study in China [36]. A Malaysian study found higher education levels, professional and managerial occupations, and higher income groups were associated with higher WTP [35]. An Indonesian study found that higher income and high perceived risk among healthcare workers were associated with higher WTP [38]. Good self-rated health status and perceived effectiveness of the vaccine for prevention and control of COVID-19 were also found as significant factors for participants’ WTP for the COVID-19 vaccine. In addition, the perceived severity of the pandemic was also associated with a higher WTP. As HBM constructs were significantly associated with WTP, the HBM model should be used to inform the development of interventions to promote vaccination against COVID-19 as a priority for expenditure.

Over 40% of the participants who intended to receive a COVID-19 vaccine wanted to get vaccinated as soon as possible. Studies conducted in China and India found people’s intention to get prompt COVID-19 vaccination was 52.5 and 65.8% respectively [24, 29]. The majority of vaccine intent participants reported that both types of vaccine (domestically-made or imported) were acceptable, while the imported vaccine was more frequently preferred compared to the domestically-made (22.9% vs 14.8%) in contrast to a study in China which found that the majority of participants preferred a domestically-made vaccine over foreign-made (64.2% vs 11.9%) [36].

Our findings suggest that information about the safety and efficacy of the COVID-19 vaccines should be made public on a regular basis and timely health education and communications by public health and government sources such as healthcare professionals are critical to alleviating public concerns as well as improving confidence and compliance with the COVID-19 vaccine [23, 53].

There are some limitations to the current study that need to be considered when interpreting the results. Firstly, this study is a cross-sectional study design that cannot establish causal inferences. Secondly, the responses were based on self-reporting and may be subject to self-reporting bias and a tendency to report socially desirable responses. Thirdly, the use of an online survey and convenience sampling may result in sampling bias, so results may not apply to the wider community due to a lack of representative samples. Finally, the study was hypothetical in nature as it was conducted before the COVID-19 vaccine became available in Bangladesh, so results may now differ in practice. However, we believe that we have captured some really important information about the COVID-19 vaccine. Further research is needed to gather more data about the COVID-19 vaccine and WTP since over 9.9 million doses of the COVID-19 vaccine have been given in Bangladesh as of June 1, 2021 [18].

## Conclusion

This study reflected that a sizeable proportion of Bangladeshi people intended to receive a COVID-19 vaccine. Low perceived susceptibility to being infected with COVID-19, as well as concern about side effects, and the safety of any new vaccine were identified as key factors in people's unwillingness or hesitation to receive a vaccine. Furthermore, the majority of participants had a willingness to pay for a COVID-19 vaccine. This study has important implications for facilitating public health and government authorities to design and deliver targeted intervention programs to enhance public acceptance of the COVID-19 vaccination in Bangladesh.

## Supplementary Information

**Additional file 1.**

## Data Availability

The datasets used and/or analyzed during the current study are available from the corresponding author on reasonable request.

## Abbreviations

COVID-19: Coronavirus disease 2019
SARS-CoV-2: Severe Acute Respiratory Syndrome Coronavirus 2
WTP: Willingness to pay
HBM: Health belief model
WHO: World Health Organization
BDT: Bangladeshi Taka
US$: United States Dollar
aOR: Adjusted Odds Ratio
OR: Odds Ratio
